# Research progress on the application of Shenque acupoint in moxibustion therapy for chronic heart failure: A review

**DOI:** 10.1097/MD.0000000000041654

**Published:** 2025-02-28

**Authors:** Ning Lv, Yingqiu Wang, Rui Hu, Yue Yang

**Affiliations:** a Heilongjiang University of Traditional Chinese Medicine, Harbin, Heilongjiang Province, China; b Heilongjiang University of Traditional Chinese Medicine, Harbin, Heilongjiang Province, China; c Department of Cardiology, The First Affiliated Hospital of Heilongjiang University of Traditional Chinese Medicine, Harbin, Heilongjiang Province, China.

**Keywords:** chronic heart failure, moxibustion therapy, research progress, Shenque acupoint

## Abstract

Because of its special characteristics, Shenque acupoint plays a pivotal role in the operation of human meridians and qi, and moxibustion at the Shenque acupoint is a good prescription for treating all diseases and strengthening the body. In this paper, a review of the research published in recent years on the application of moxibustion at the Shenque acupoint in patients with chronic heart failure (CHF) was carried out, and the authors found that supplementing moxibustion at the Shenque acupoint with conventional Western medical treatment, regardless of whether it is a single point of moxibustion at the Shenque acupoint, with multiple points of moxibustion therapy, or combined with other therapies, has achieved significant results in relieving the clinical symptoms of patients with CHF, improving cardiac function, and improving quality of life. Therefore, the Shenque acupoint has an important position in the selection of moxibustion points for patients with CHF, is easy to locate and convenient to operate, and is worth being widely used in the clinic. However, the related mechanism research has not yet been deepened and focused, and the clinical study still lacks a strict methodological design; therefore, future research prospects are far from reaching.

## 1. Introduction

**Figure 1.**
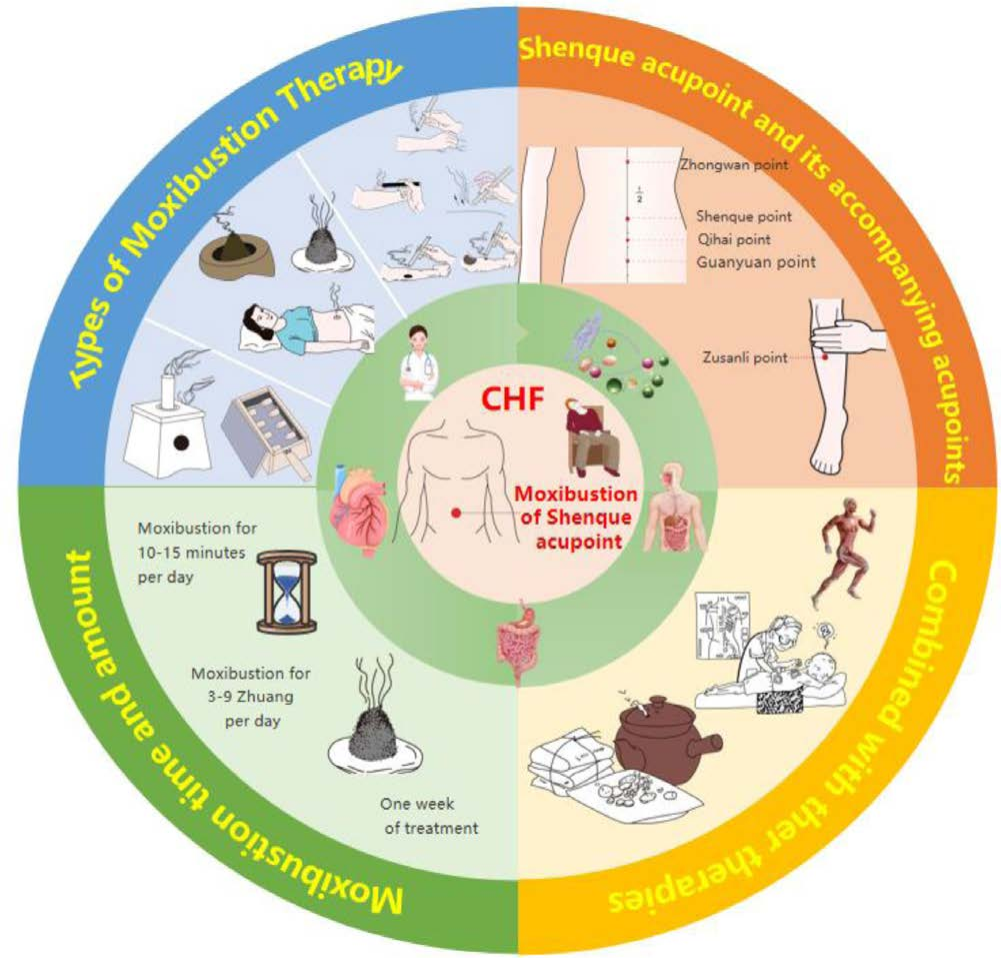
Moxibustion of Shenque acupoint. CHF = chronic heart failure.

Chronic heart failure (CHF) is a clinical syndrome characterized by circulatory congestion with symptoms such as dyspnea, asthenia, and edema.^[1]^ It represents the most severe condition in the progression of various cardiovascular diseases. Currently, treatment in Western medicine primarily involves medications such as cardiotonics, diuretics, and vasodilators. Although the medications have achieved significant progress in the treatment of CHF, their effectiveness in improving quality of life and long-term prognosis remains unsatisfactory.^[2]^ As the state vigorously supports the application of traditional Chinese medicine (TCM) nursing techniques in clinical practice, the State Administration of Traditional Chinese Medicine officially promulgated and implemented the “TCM Nursing Programme for 13 Disease Types (Trial)”^[3]^ in 2013, including the “TCM Nursing Programme for Heart Failure Disease” (hereinafter referred to as the “Program”). Moxibustion therapy, as one of the TCM nursing treatments in the Program, has been widely used in the adjuvant treatment of CHF and has achieved good efficacy and patient recognition.^[4,5]^ In addition, the mechanisms of moxibustion in improving heart function and delaying the progression of CHF have been verified in multiple experimental studies.^[6–9]^ After reviewing the extant literature (Supplementary File 1, Supplemental Digital Content, http://links.lww.com/MD/O420), it was found that the Shenque acupoint is frequently involved in moxibustion therapy for CHF (Fig. 1). Therefore, in-depth research on this aspect was conducted and illustrated as follows.

The Shenque acupoint, first mentioned in the “Huangdi Neijing” as “Qishe” or “Qihe,” is known for its unique location at the navel, which has direct or indirect connections with the Chong, Ren, Du, and Dai meridians among the “Eight Extraordinary Meridians.” Through connections with the “Eight Extraordinary Meridians” and the “Twelve Meridians,” the Shenque acupoint indirectly regulates and harmonizes qi, blood, and meridians throughout the body.^[10]^ According to Western medical studies, the navel area is one of the last to develop and close after birth and has a recessed appearance with few subcutaneous fats.^[11]^ In particular, abundant blood vascular networks and neural networks are present in the area, including the paraumbilical vein, superficial thoracoepigastric veins, intercostal veins, and 10th and 11th branches of the thoracic nerves.^[12]^ Additionally, as the Shenque acupoint is anatomically unique, the percutaneous administration of medication at this acupoint is accompanied by rapid drug absorption rates, which lead to high requirements for drag permeability at the Shenque acupoint.^[13]^ Therefore, the position of the Shenque acupoint contributes to its pivotal effects.

## 2. Overview of Shenque acupoint moxibustion therapy

**Figure 2.**
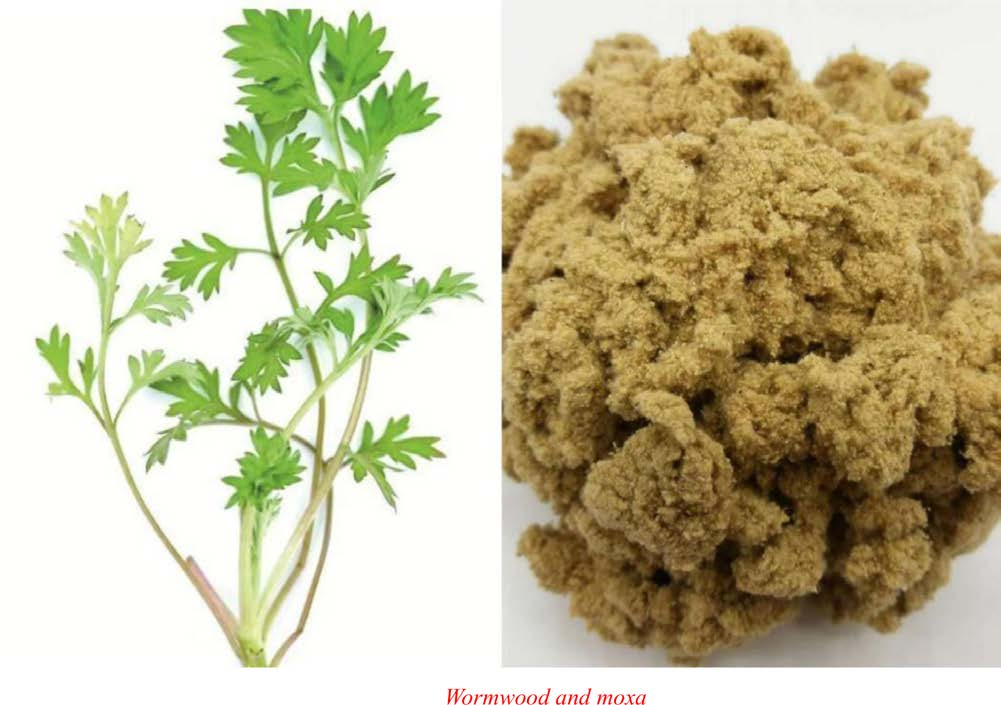
Wormwood and moxa.

Moxibustion therapy is a treatment to warm a combination of acupoints with materials consisting of moxa wool,^[14]^ as shown in Figure 2. According to “Yi Bei Lu” (Divergent Views in Medicine), moxa is bitter, mildly warm, nontoxic, and primarily used to treat a variety of diseases. Hence, burning moxa strengthens yang qi. Furthermore, moxibustion aims to achieve therapeutic and health benefits by stimulating the acupoints. As stated in “Yi Xue Ru Men” (Introduction to Traditional Chinese Medicine), moxibustion must be applied where drugs and needles fail to cure. Similarly, in “Bian Que Xin Shu” (Bian Que’s Book on the Heart) on major diseases appropriate for moxibustion, it is recorded that for all major diseases, moxibustion therapy should be applied below the navel (Shenque acupoint) with 500 zhuang of moxa cones to replenish genuine qi. Accordingly, the following representational instructions are found in “Jia Yi Jing” (A-B Classic of Acupuncture). First, it is forbidden to needle the center of the navel, as it will cause ulcers, and patients who further develop symptoms of incontinence would die irremediably. Second, for infertility, moxibustion was applied to the center of the navel area to conceive. Third, for symptoms of edema and flat navel, moxibustion was applied to the center of the navel. As a result, the Shenque acupoint is crucial in the treatment of tonifying the kidney, strengthening the biological function of the spleen and unblocking the bowel, regulating qi and resolving stagnation, astringing the intestine and stopping diarrhea, tonifying qi to prevent collapse and secure semen, and stopping leukorrhea. As a result, moxibustion at the Shenque acupoint is a major and effective therapy for curing various diseases and strengthening the body.^[15]^ Meanwhile, notable results have been found in previous experimental studies. The application of moxibustion therapy at the Shenque acupoint in long-term exhausted rats could improve its antioxidant capacity, alleviate myocardial damage, relieve fatigue, and increase the number of immunoglobulins in rats.^[16–18]^ Moreover, moxibustion at both the Shenque and Zusanli acupoints could intervene in the occurrence of exercise fatigue and improve cardiac function more effectively.^[19]^

## 3. Analysis of the characteristics of clinical application of moxibustion at Shenque acupoint for CHF patients

### 3.1. Types of intervention for CHF with moxibustion at Shenque acupoint

According to the diversity of the clinical applications of moxibustion, moxa stick moxibustion, moxa cone moxibustion, warm needle moxibustion, and so on. Each category has many specific and different clinical applications, and each type of moxibustion therapy has its own characteristics. Moxa stick moxibustion and moxa cone indirect moxibustion are currently used for patients with CHF. This is probably because these 2 types of moxibustion are gentle, simple to use in clinical procedures, highly accepted by patients, and easy to promote and implement.

#### 3.1.1. Moxa stick suspended moxibustion

**Figure 3.**
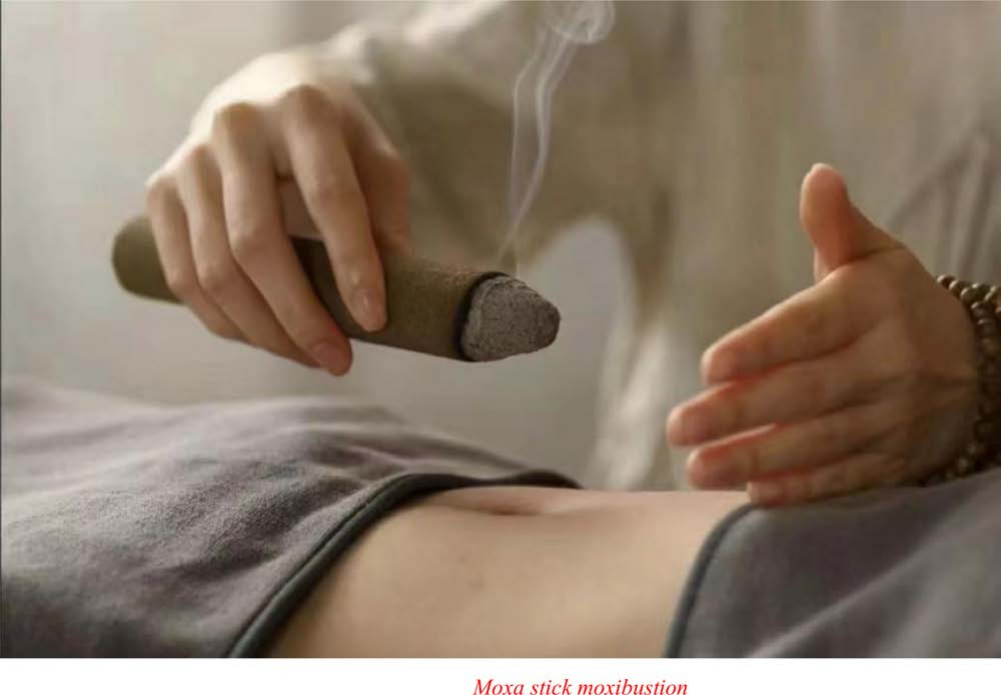
Moxa stick moxibustion.

Suspended moxibustion is the main operating technique of moxa stick moxibustion,^[20]^ as shown in Figure 3. It can be further classified into gentle moxibustion, sparrow-pecking moxibustion, and circling moxibustion.^[21]^ The technique involves suspending a moxa stick a certain distance from the skin over an acupoint, causing continuous smoldering that generates consistent warm stimulation of the partial skin.^[20]^ First, patients with CHF usually choose gentle moxibustion for the intervention. A moxa stick was suspended at a fixed point with a distance of 1 cun (taking the width of the thumb as 1 cun) over the acupoints. Through continuous heat instead of burning stimulation, it can warm and regulate qi and blood and reinforce healthy qi to eliminate pathogenic factors.^[22,23]^ According to a study with a sample of 100 patients with CHF (Zhou et al),^[24]^ applying gentle moxibustion at the Shenque acupoint and the adjunctive acupoints could drastically improve cardiac function and reduce BNP and CA125 levels. Similarly, Fan et al^[25,26]^ stated that this technique could alleviate gastrointestinal discomfort and improve quality of life. All the above effects were found by Douxuan^[27]^ and Xia et al.^[28]^ They combined the technique with the application of compound clove appetizer patches and self-made intestinal patches at the Shenque acupoint and adjunctive acupoints. Furthermore, combining gentle moxibustion, sparrow-pecking moxibustion, and circling moxibustion could strengthen the qi of meridians while warming the body, circulating qi and blood, and achieving better results. Huanfeng et al^[29]^ conducted research on the combination of techniques for 15 to 20 minutes in each session. The participating patients with CHF showed relief from constipation and a reduction in the incidence of adverse cardiac events. However, this combination therapy is rarely applied and requires further research. Additionally, although gentle moxibustion is mild, the expected results may not be achieved in clinical studies. Effective compensation is heat-sensitive moxibustion, under the category of moxa stick moxibustion. To perform the therapy, different moxa sticks are applied to heat-sensitive points that are identified through suspended moxibustion.^[30]^ This technique was demonstrated by Shanshan et al^[31]^ with a sample of 50 patients with CHF who were resistant to diuretics. The patients were evenly divided into a control group, in which they received standard Western medical treatment, and an experimental group. The latter additionally received heat-sensitive moxibustion therapy from the Shenque acupoint to the Huiyin acupoint at the lower abdominal and surrounding areas. After 14 days, the effective rate in the experimental group with TCM syndrome treatment was 96%, which was considerably higher than that in the control group (72%). Improvements, such as an increase in urine output, exercise tolerance, and diuretic resistance without causing renal function abnormalities or electrolyte disturbances, indicate the higher effectiveness of heat-sensitive moxibustion in improving clinical symptoms. Other benefits, including promoting the recovery of cardiac function, enhancing the clinical effects on edema and abdominal distension, and improving the quality of life, have also been reported by Lan et al.^[32]^ Haibin et al^[33]^ combined heat-sensitive moxibustion with oral administration of intestinal lubricant pills and found that the combined treatment could help patients by regulating the function of zang-fu, alleviating constipation symptoms, and increasing patient compliance.

#### 3.1.2. Moxa cone indirect moxibustion

Moxa cone indirect moxibustion involves forming moxa wool into a cone shape, placing it on top of slices of ginger, garlic, or cake of fork medicines, and kindling them. The intense heat and efficacy of Chinese medicines help to penetrate more deeply, making the effects stronger than those of gentle moxibustion. Common techniques for patients are ginger-insulated moxibustion and medicine-insulated moxibustion; the latter is further classified as navel moxibustion and flour bowl moxibustion.

**Figure 4.**
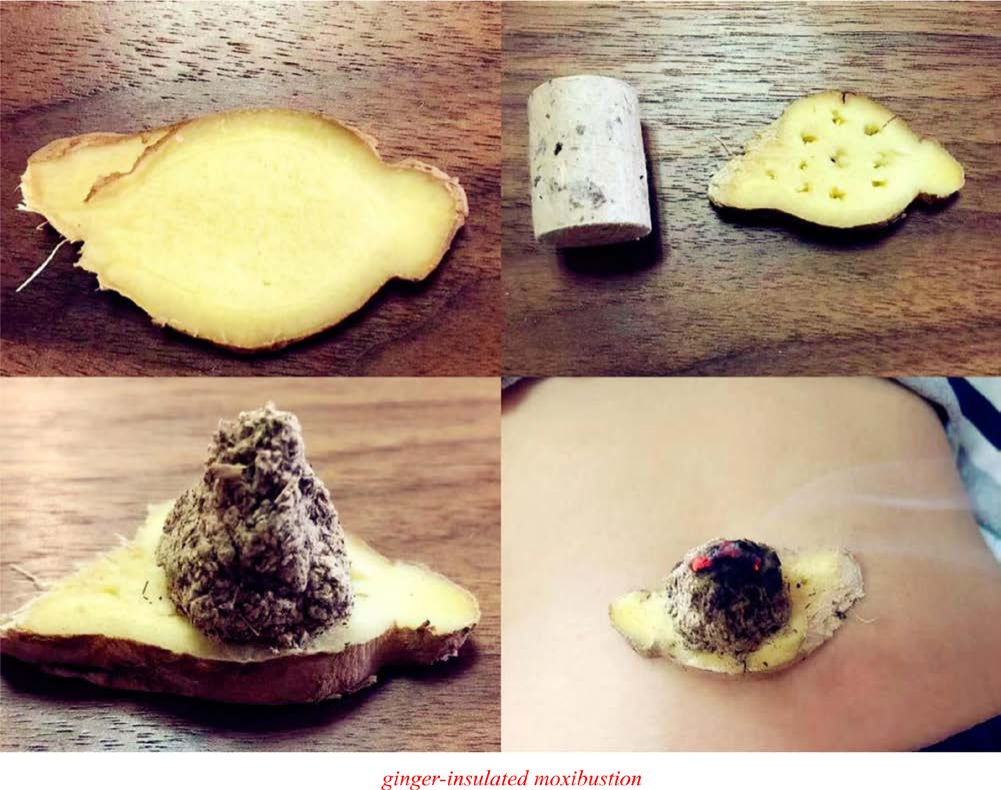
Ginger-insulated moxibustion.

First, ginger-insulated moxibustion is most frequently applied in clinical practice, where a ginger slice serves as a medicinal pad between the moxa cone and acupoint, leveraging the efficacy of both Chinese medicine and moxibustion,^[34]^ as shown in Figure 4. Related research with a sample of 62 CHF patients was undertaken by Zhennan^[35]^ and designed an experimental group operating ginger-insulated moxibustion under regular treatment and care; for a 2-week course, patients received therapy with 3 zhuang of moxa cones once a day, in which the dosage was equivalent to a 15-minute session. Ginger-insulated moxibustion not only performs better than regular care in improving gastrointestinal symptoms but is also safe, uncomplicated, and convenient. Xiangyi et al^[36]^ studied ginger-separated moxibustion at the Shenque acupoint and adjunctive acupoints with alterations. Patients were instructed to lift the ginger slice or replace the moxa cone if they felt localized burning pain during moxibustion and prescribed Simo decoction as a combined treatment. The approach is therapeutically effective and could assist patients with CHF in correcting intestinal flora imbalances, suppressing inflammatory responses, improving exercise endurance, and enhancing abilities in daily living. Similarly, Lisa et al^[37]^ set the dosage of moxa cones to 9 zhuang with replaced ginger slices in each of the 3 zones of moxa cones. While applying ginger-insulated moxibustion at the Shenque acupoint, they integrated the acupoint application at other acupoints. After 2 weeks, it was observed that 92.5% of CHF patients in the experimental group showed progress in their health condition, while only three-quarters of the CHF patients in the control group achieved the same result, demonstrating that this combined approach is an effective treatment for CHF. Another commonly applied method is navel moxibustion, which is essentially to apply medicine-insulated moxibustion at the navel (Shenque acupoint). It utilizes the epidermal attributes of the navel, such as low thickness, high sensitivity, and quick absorption rate, and the yang heat produced during burning moxa to penetrate medications through the navel skin and stimulate tissues, thereby regulating and harmonizing qi and blood, unclogging the meridians, preventing diseases, and maintaining fitness. Chao et al^[38]^ studied navel moxibustion in combination with Zhenwu decoction. To start the therapy, a dough ring was placed on the navel, the dough was filled with the powder made from ephedra, asarum, and sinapis alba in a ratio of 2:1:1, flattened conical moxa cones were placed, and moxibustion was performed at the Shenque acupoint until the moxa was consumed. The dough ring was removed, the powder was covered with medical tape, and the navel was cleaned for 2 hours. After a course of 4 weeks, the experimental group had a total effectiveness rate of 90.00% (27/30), outperforming the control group, with a total effectiveness rate of 73.33% (20/30). It has significantly improved the inflammatory and oxidative stress responses in patients with CHF and consequently delayed the onset and progression of CHF.

**Figure 5.**
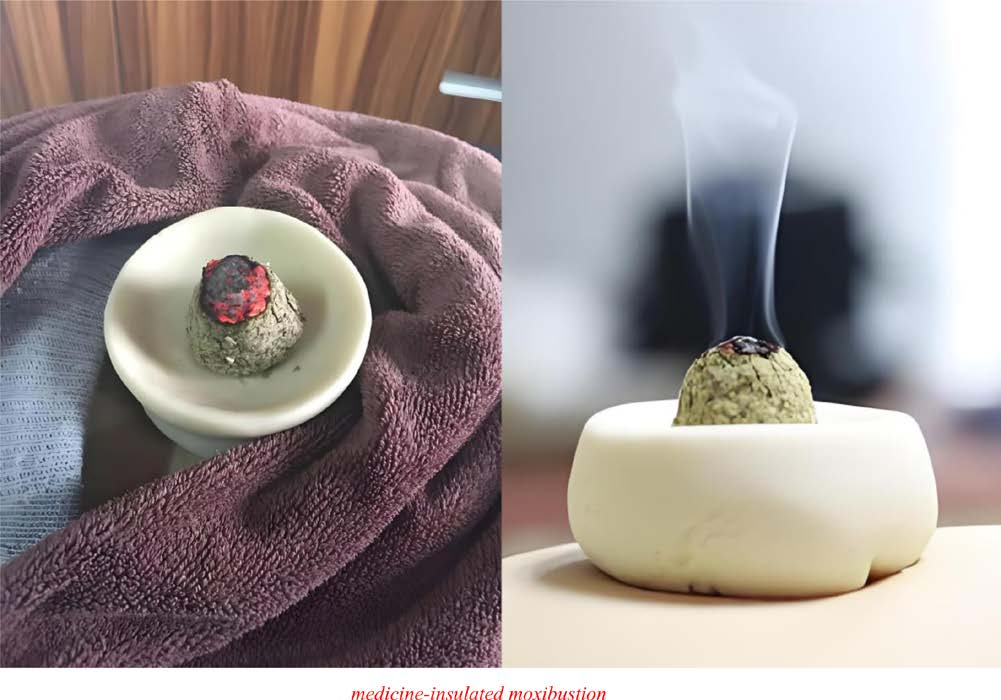
Medicine-insulated moxibustion.

Moreover, a particular technique of navel moxibustion is flour bowl moxibustion. Similar to the above study, the procedure starts by placing a prepared flour bowl on the navel area (Shenque acupoint), then filling the bowl with powder and kindling the moxa applied over the navel,^[39]^ as shown in Figure 5. As flour has a tonifying effect, the flour bowl can regulate the spleen and stomach. Its sealed nature enhances its effectiveness at the Shenque acupoint, preventing “medicinal qi” from escaping. Compared to other techniques, flour bowl moxibustion combines the effects of a flour bowl, acupoints, and Chinese medicines, thereby offering multiple benefits.^[40]^ In a study with a sample of 120 patients with CHF, Hongli et al^[39]^ randomly divided them into an experimental group and a control group with 60 patients in each group. The control group received standard Western medical treatment, whereas the experimental group received a combination of Zhenwu decoction and flour bowl moxibustion at the Shenque acupoint. The therapy was applied once a day for one and a half hours (6 Zhuang moxa cones). After 7 days, it was evident that the efficacy was better in the experimental group, the N-terminal pro-B-natriuretic peptide level was regulated, and the approach was safe and reliable.

#### 3.1.3. Modern moxibustion devices

**Figure 6.**
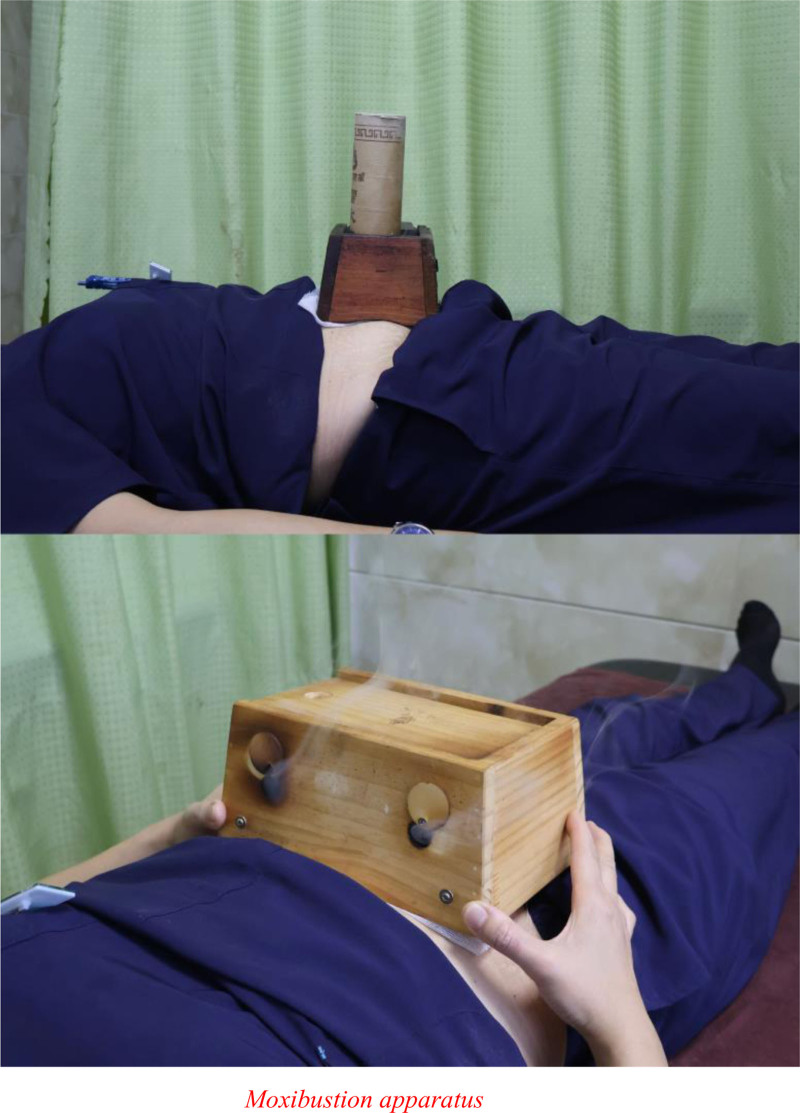
Moxibustion apparatus.

The modern practice of moxibustion often involves moxibustion boxes and similar devices, where moxa wool is ignited inside the device, and the bottom nonwoven mat is placed at the acupoint for ease of operation and higher patient acceptance, as shown in Figure 6. Moxibustion boxes were examined in a large amount of research. For instance, Nengzong^[41]^ applied it to the Shenque acupoint. Wenyan^[42]^ applied it at the Shenque and adjunctive acupoints. Fu et al^[43]^ combined moxibustion with exercise and a 7-step rehabilitation program. Research findings consistently showed that involving a moxibustion device in the therapy could significantly optimize the treatment effects, alleviate symptoms in patients with CHF, enhance cardiac function, and improve quality of life. Therefore, among the moxibustions that use the Shenque acupoint to intervene in CHF, the most common is gentle moxibustion, followed by moxa cone ginger-insulated moxibustion. Both approaches could noticeably improve clinical efficacy, regulate cardiac function indicators, enhance cardiac function, and improve the quality of life, making them clinically meaningful and worthy of wide clinical adoption. While other moxibustions, such as heat-sensitive moxibustion, navel moxibustion, and flour bowl moxibustion, have unique advantages, their clinical application is less common, and related research is required.

### 3.2. Duration and dosage of moxibustion at Shenque acupoint for CHF intervention

The moxibustion duration included the length of time, frequency, and course of treatment. According to “Bian Que Xin Shu” (Bian Que’s Book on the Heart), for major diseases, apply 100 strong moxa cones, for minor diseases, applying a few is enough.^[15]^ This suggests that the quantity and intensity of moxibustion should be tailored according to the patient’s constitution, age, condition, and body area. Meanwhile, among the research studies on moxibustion at Shenque acupoint for CHF intervention, it was found that the duration for each session is typically between 10 and 20 minutes, and patients take 1 session each day with a course lasting for 1 week. Limited research on moxa cone moxibustion has discussed moxibustion dosage, ranging from 3 to 9 zhuang per day. This indicates the need for further research in this aspect.

### 3.3. Comparison between the application of the Shenque acupoint alone and with adjunctive acupoints for CHF intervention

In moxibustion therapy applied at the Shenque acupoint for patients with CHF, some studies only used the Shenque acupoint, while others used a combination of the Shenque acupoint and other acupoints. Comparing the 2 methods revealed that single acupoint treatment primarily improves gastrointestinal symptoms,^[35,41,44,45]^ while multi-acupoint treatment relies on the following selection pattern to obtain different effects, to improve symptoms such as vomiting, abdominal distension, and constipation, and to enhance survival quality; the most common selection is the Shenque acupoint combined with the Zhongwan acupoint, Zusanli acupoint, and others. For instance, Lingling et al^[46]^ selected the Shenque, Zhongwan, Double Zusanli, and Weishu acupoints. Moreover, Fan et al^[25,26]^ chose the Shenque, Zhongwan, Double Zusanli, Double Neiguan, and other acupoints. Both studies demonstrated effectiveness in alleviating gastrointestinal discomfort and improving survival in patients with CHF. Second, to improve the symptoms of edema and enhance cardiac function, common patterns include the Shenque, Zhongwan, Guanyuan, Shuifen, and Fenglong acupoints. For instance, Wenyan^[42]^ selected the Shenque, Zhongwan, Guanyuan, Qihai, and Shuifen acupoints; Lan et al^[32]^ applied the Shenque, Zhongwan, Guanyuan, Fenglong, and Zusanli acupoints; and Zhou et al^[24]^ chose the Shenque, Zhongwan, Guanyuan, and Qihai acupoints. All patterns effectively improved edema and cardiac function in patients with CHF. Therefore, the prioritized adjunctive points were ordered as Zhongwan, Guanyuan, Qihai, and Zusanli acupoints. The Ren meridian passes through the Zhongwan, Guanyuan, and Qihai acupoints. These acupoints could be used to nourish qi and blood and to support the liver and kidneys. The Zhongwan acupoint is the crossing point between the Ren meridian and meridians, including the small intestine meridian pattern, Sanjiao meridians, stomach meridian, and the front-mu point of the stomach. It could potentially strengthen the spleen and stomach, transform the phlegm, and resolve stagnation. The Guanyuan acupoint reinforces healthy qi to strengthen the body and supplement qi and tonify yang. The Qihai acupoint warms and tonifies the lower jiao, benefits the essence of the kidney, supplements qi, and calms the mind. The Zusanli acupoint strengthens the spleen and stomach, supplements the Qi of the middle jiao, regulates Qi and blood, and unclogs the meridians. Through comparison, it was found that the combination with adjunctive acupoints is more effective in improving clinical symptoms, enhancing cardiac function, and strengthening quality of life, and performs better for CHF patients in optimizing the circulation of Qi and blood throughout the body. However, a single acupoint application stands out for improving gastrointestinal symptoms. Although its efficacy is limited, it is both straightforward and easy to implement. After passing the acute and dangerous phase in the hospital, patients who require long-term home care can receive Shenque acupoint moxibustion therapy at home with professional guidance. It can prolong the life cycle of patients with CHF and reduce the pressure of both doctors and patients, but how to guide patients to apply moxibustion, how to improve patients’ adherence to home moxibustion therapy, and how to dynamically assess the efficacy of home moxibustion therapy for patients still need to be studied in depth by more scholars.

### 3.4. Comparison of the application of Shenque acupoint moxibustion therapy alone and with adjunctive therapy to treat CHF

Combining other therapies involves the use of Shenque acupoint moxibustion with Chinese herbal decoctions, Chinese herbal pills, acupoint application, and exercise. Chinese herbal decoction is most commonly used in clinics, with noteworthy effects in suppressing inflammatory responses. For instance, Chao and Dandan^[38]^ combined Zhenwu decoction with navel moxibustion and showed nonnegligible results for patients with CHF in ameliorating inflammation and oxidative stress, thereby delaying the onset and progression of CHF. Similarly, Xiangyi et al^[36]^ combined Simo decoction with ginger-insulated moxibustion and found it helpful in correcting intestinal flora imbalances, suppressing inflammatory responses, and enhancing the abilities of daily living in patients with CHF. Combined exercise could further enhance the endurance of patients. Moreover, Fu et al^[43]^ studied the application of a moxibustion box over the Shenque acupoint in adjunctive therapy, combining it with a 7-step exercise rehabilitation program. Four weeks later, coronary heart disease patients with heart failure showed improved heart failure indicators and enhanced exercise endurance, thereby improving their quality of life. In addition, the effectiveness of other combined therapies^[27,28,33,37]^ cannot be distinguished from the application of moxibustion therapy alone. Applying any method could improve clinical symptoms and regulate cardiac function indicators, thereby enhancing cardiac function, improving quality of life, and delaying the onset and progression of CHF.

## 4. Conclusion

Through reviewing the research of moxibustion therapy at the Shenque acupoint, moxibustion duration and dosage, comparative use of single and multiple acupoints, and comparison of moxibustion at the Shenque acupoint combined with other therapies, the following results have been found. First, moxibustion therapy commonly uses gentle moxibustion to achieve a comprehensive effect. Second, the course of moxibustion is commonly 1 week with 1 session each day and 10 to 20 minutes in each session. Few studies have included the moxibustion dosage, with the exception of moxa cone moxibustion. The suggested dosage varies from 3 to 9 zhuang of moxa cones per day. Third, single acupoint moxibustion at the Shenque acupoint performed the best in improving gastrointestinal symptoms, although its effectiveness in other aspects was not obvious. On the other hand, multi-acupoint moxibustion at the Shenque acupoint and adjunctive acupoints has broader effects, including improving symptoms in the gastrointestinal tract and edema, enhancing cardiac function, and improving the quality of life. Acupoint selection commonly involves the Zhongwan, Guanyuan, Qihai, and Zusanli acupoints. Fourth, the most commonly applied treatment involves combining Shenque acupoint moxibustion with Chinese herbal decoctions, which are notably effective in suppressing inflammatory responses. Other combined treatments showed no significant differences compared with moxibustion alone. Overall, for patients with CHF, supplementing the conventional Western medicine treatment with Shenque acupoint moxibustion therapy, such as the single acupoint moxibustion at the Shenque acupoint, moxibustion therapy at the Shenque acupoint with additional acupoints, and moxibustion therapy at the Shenque acupoint with other therapies, could achieve considerably more effective results, which can significantly improve the clinical symptoms of patients with CHF and improve cardiac function and quality of life.

TCM categorizes CHF under “palpitations,” “edema,” and “cardiac water,” viewing it as a chronic disease mixed with deficiency and excess. It is characterized by the primary pathogenesis of yang(qi) deficiency of the heart and kidney, water fluid, and blood stasis retention. According to the pathogenic characteristics, the treatment principle should focus on tonifying yang and supplementing qi, draining water retention, and circulating blood. The Shenque acupoint, being an inborn acupoint of the Ren meridian, is located between the middle and lower burners, where the original qi is long-preserved. Internally, the acupoint connects the 12 meridians, 5 viscera, 6 bowels, and limbs and bones, facilitating the circulation of all meridians.^[47]^ Moxibustion at the Shenque acupoint can effectively improve the symptoms in heart failure patients, warm and harmonize the heart and kidneys, transform qi and regulate water, and invigorate the circulation of blood and resolve stasis. Therefore, we believe that in the treatment of CHF with moxibustion therapy, the Shenque acupoint is very important. The therapy is safe, convenient, and can be applied clinically.

## 5. Limitations and future prospects

Currently, there are shortcomings in the related research. The following limitations were found by organizing and summarizing the literature.

First, although there is a certain pattern in the selection of acupoints for moxibustion therapy at the Shenque acupoint with additional acupoints, a unified instruction has not yet been formulated, and standardization of the selection and application of acupoints and development of uniform standards is a future research direction.

Second, clinical studies lack a rigorous methodological design, and there is a lack of multicenter, large-sample relevant studies, which cannot strongly illustrate the therapeutic effect of moxibustion of the Shenque acupoint on CHF. Meanwhile, the mechanism of moxibustion at the Shenque acupoint should be studied in depth in the future, and animal experimental studies can be carried out to elucidate the relationship between the Shenque acupoint and CHF and prove the effectiveness of the Shenque acupoint in moxibustion treatment of CHF from the level of the mechanism is the focus of moxibustion treatment research in the future.

Third, in patients with CHF, although moxibustion at the Shenque acupoint is more effective, its use is currently limited to the hospitalization period, and most patients still require long-term home application of moxibustion after discharge. In the future, we can extend moxibustion therapy to the community and home based on mobile healthcare and the triadic linkage care model of hospitals, communities, and homes to provide continuity of care for patients with CHF to improve the quality of survival and prolong the life cycle of patients, which is an important direction of the study and will be a blessing for patients with CHF.

Fourth, the quality of the clinical research literature is mixed, and further verification of its internal authenticity is needed. Moreover, most articles did not explicitly mention the type of study, the sample size of the study was small, and the undeniable occurrence of selection bias, measurement bias, publication bias, etc, resulted in a low level of evidence. In the future, we hope that more scholars will conduct large-scale primary research or secondary research on data (e.g., moxibustion time and moxibustion volume) through evidence-based medicine so as to provide strong evidence for clinical implementation.

## Author contributions

**Visualization:** Ning Lv.

**Writing – original draft:** Ning Lv.

**Writing – review & editing:** Ning Lv, Yue Yang.

**Validation:** Ning Lv, Yue Yang.

**Funding acquisition:** Yue Yang.

**Supervision:** Yue Yang.

## Supplementary Material


