# Supplementary material for: Research progress on the application of Shenque acupoint in moxibustion therapy for chronic heart failure: A review
Source: Medicine (Baltimore). 2025 Feb 28;104(9):e41654. doi: 10.1097/MD.0000000000041654 (PMC11875596; doi:10.1097/MD.0000000000041654)
Supplement: Supplementary file 1 [file medi-104-e41654-s001.doc]

Supplementary File 1  Although the key term of this paper is "moxibustion at the Shenque acupoint", during the literature - searching process, it is inadequate to merely search for "the Shenque acupoint". To ensure a more comprehensive literature search, it is essential to include both "heart failure" and "moxibustion" in the search scope.

Taking the PubMed database as an instance, the search pattern is presented as follows：

（＂Moxibustion" [MeSH Terms] OR "Moxibustion"[Title/Abstract])AND ("Heart Failure"[MeSH Terms] OR ("cardiac failure"［Title/Abstract] OR "heart decompensation"[Title/Abstract] OR＂decompensation heart" [Title/Abstract] OR "congestive heart failure" [Title/Abstract] OR "heart failure congestive"［Title/Abstract] OR "heart failure right sided"[Title/Abstract]OR "heart failure right sided " [Title/Abstract] OR "right sided heart failure" [Title/Abstract] OR "right sided heart failure"［Title/Abstract] OR "heart failure left sided"[Title/Abstract]OR "heart failure left sided" [Title/Abstract] OR "1eft sided heart failure" [Title/Abstract] OR"left sided heart failure［Title/Abstract] OR "myocardial failure"[Title/Abstract]))


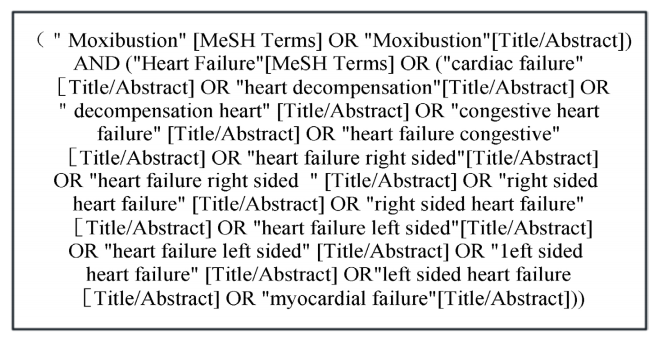


PubMed retrieval mode
